# Anaplasmosis Outbreak in Lambs: First Report Causing Carcass Condemnation

**DOI:** 10.3390/ani10101851

**Published:** 2020-10-12

**Authors:** Delia Lacasta, Luis Miguel Ferrer, Santiago Sanz, Raquel Labanda, José María González, Alfredo Ángel Benito, Héctor Ruiz, Ana Rodríguez-Largo, Juan José Ramos

**Affiliations:** 1 Animal Pathology Department, Instituto Agroalimentario de Aragón-IA2 (Universidad de Zaragoza-CITA), Veterinary Faculty of Zaragoza, C/Miguel Servet 177, 50013 Zaragoza, Spain; lmferrer@unizar.es (L.M.F.); jmgsovino@gmail.com (J.M.G.); hectorruiz353@gmail.com (H.R.); anarlg@posta.unizar.es (A.R.-L.); jjramos@unizar.es (J.J.R.); 2 ADS Nuestra Sra, del Pueyo, C/Pilar 21, 50130 Belchite, Spain; santiagosanzv@telefonica.net; 3 Casa de Ganaderos de Zaragoza, C/San Andrés, 8, 50001 Zaragoza, Spain; raquel.cgz@hotmail.com; 4 Gabinete Técnico Veterinario S.L, C/Isla conejera sn, 50013 Zaragoza, Spain; 5 Exopol S.L. Pol. Río Gállego D-8, San Mateo de Gállego, 50840 Zaragoza, Spain; abenito@exopol.com

**Keywords:** anaplasmosis, lambs, *Anaplasma ovis*, jaundice, carcass condemnation

## Abstract

**Simple Summary:**

Ovine anaplasmosis has been described in tropical and sub-tropical countries, where it produces a mild syndrome characterised by anaemia, weakness, weight loss and drop in production; however, in recent years, this pathogen has been found in temperate countries, where it causes more severe disease. This is the first description of an outbreak of anaplasmosis in lambs causing icteric carcass condemnation at the abattoir. The authors would like to draw attention to this emerging disease that is causing significant economic losses in sheep farming.

**Abstract:**

In spring and summer 2020, six outbreaks of condemnation of jaundiced lamb carcasses were diagnosed in different farms in Aragón region, Spain. *Anaplasma ovis* was identified in all affected farms. Four hundred and ninety-two lambs from two affected farms were more closely examined. Clinical examination, haematologies, biochemistries, histopathology and microbiological and molecular analyses were performed. After slaughter, 34.84% of the lambs showed jaundiced carcasses and 79.64% presented splenomegaly at the abattoir. All tested lambs with icteric carcasses showed positive *A. ovis* PCR, although 72.72% of the unaffected lambs also tested positive. However, the bacterial load was significantly higher in the animals that showed jaundiced carcasses (Cq: 25.00 vs 26.16; *p* = 0.004). Moreover, all the tested lambs that showed severe anaemia were PCR positive. On the contrary, the PCR negative lambs did not show anaemia. Lambs that presented icteric carcasses displayed severe regenerative anaemia with significantly lower erythrocyte count (7.18 vs. 11.97), haematocrit (26.89 vs. 34.82) and haemoglobin (8.50 vs. 11.10) than unaffected lambs. Reticulocyte count (18.80 vs. 5.65) was also significantly increased in affected animals. This article describes a new disorder caused by *Anaplasma ovis* that is producing significant economic losses associated with the carcass condemnation of apparently healthy lamb.

## 1. Introduction

*Anaplasma ovis* is an obligate intraerythrocytic Gram-negative bacterium of the family Anaplasmataceae. This species is primarily transmitted by ticks and can infect sheep, goats and wild ruminants [1].

Ovine anaplasmosis has been described in tropical and subtropical countries, where it produces a mild syndrome characterised by anaemia, weakness, weight loss and drop in production [2]. However, advances in molecular diagnostic techniques have demonstrated the presence of this disease in many developed countries, such as Hungary, Portugal, Romania and Turkey [3,4]. Several studies show that the prevalence of *A. ovis* in the Mediterranean area is significant [5], although it is barely diagnosed at the field level. In Spain, ovine anaplasmosis was first described in 2014 [6], causing a severe outbreak in livestock areas in the north of the country. Adult sheep and especially young animals from 1 to 3 years old were affected. The clinical signs were severe anaemia, extreme weakness, anorexia and weight loss [6].

The main vectors of the *Anaplasma* bacteria are ticks, particularly the genera *Ixodes, Dermacentor, Rhipicephalus* and *Amblyomma* [1], although mechanical transmission can be produced by fleas, tabanids, and even needles and other veterinary instruments [7,8]. It should also be noted that the coexistence of wild and domestic ruminants in a temperate environments favours the activity of ticks and a high prevalence of the disease [9].

Within the erythrocyte, the bacterium replicates by binary fission to form up to eight individual organisms within a simple vacuole, and leaves it using a not-well-defined mechanism, but apparently not lytic, to infect new erythrocytes [10]. During the acute phase, the number of infected erythrocytes doubles every 24–48 h [11]. The destruction of the erythrocytes and the consequent anaemia are not, however, immediate, beginning to show noticeable changes around 30–40 days after infection [6]. The haemolytic anaemia associated with this disease is a consequence of the immune response that causes anaplasma in the body. After antigen presentation, CD4 + Th1 T lymphocytes produce IFN-γ, inducing the production of IgG2 which, in coordination with activated macrophages, are capable of destroying infected erythrocytes due to opsonisation and nitric oxide production, respectively [12].

The main clinical sign of ovine anaplasmosis is anaemia resulting from the destruction of erythrocytes. However, as it is a long-lasting process, signs such as jaundice or haemoglobinuria have not been associated with this disease [1,6]. Likewise, the disease has usually been diagnosed in adult sheep and has not been reported in lambs. The present work is the first description of an anaplasmosis outbreak in apparently healthy fattening lambs whose carcasses were condemned at the slaughterhouse for presenting jaundice.

## 2. Materials and Methods

During spring 2020, six clinical cases of icteric carcasses were referred to the Ruminant Clinical Service of the Veterinary Faculty of Zaragoza, Spain. They came from different areas of Aragón (Spain), and showed exactly the same clinical signs—apparently healthy lambs whose carcasses were condemned due to their intense yellow colouration after slaughter.

In the first step, the possibility that carotenoid pigments were staining the carcass was assessed by introducing a small piece of yellow fat into alcoholic and ether solutions. Subsequently, clinical examination, haematological, biochemical, histopathological, microbiological and molecular analysis were performed. The content of ten intestinal samples collected after slaughter was analysed by quantitative PCR analysis for detection of *Clostridium perfringens* and recognition of *C. perfringens* toxins was carried out by means of an ELISA test. These samples were collected from lambs showing intense yellow intestinal content. Further, real-time PCR of blood samples were performed for the main hemoparasites: *Babesia* sp., *Theileria* sp., *Mycoplasma ovis* and *Anaplasma* sp. After presumptive diagnosis of anaplasmosis, the lambs from two of the affected farms were examined more closely.

The care and use of animals was performed in accordance with the Spanish Policy for Animal Protection RD53/2013, which meets the European Union Directive 2010/63 on the protection of animals used for experimental and other scientific purposes.

### 2.1. Studied Lambs

All affected farms were meat farms managed under a semi-intensive production system, with stabling of the animals at the end of gestation and during the beginning of lactation, and grazing for the rest of the year. Although the dams grazed outdoors, especially in springtime, lambs were always kept indoors until slaughter.

Four hundred and ninety-two lambs from two affected farms were examined one or two days before slaughtering throughout the five weeks that the study lasted. Two hundred and forty-four lambs belong to Farm A, and two hundred and forty-eight came from Farm B. Both farms raise Rasa Aragonesa sheep and produce lambs with protected geographical indication (PGI) “Ternasco de Aragón” (two- to three-month-old lambs with 21 to 23 kg of live weight). Farm A has 2300 sheep and is located in the centre of Aragón at 600 m above sea level with an average annual rainfall of around 350 mm per year. It follows a STAR reproductive management system, with five annual mating periods lasting 22 days. Farm B has 1000 sheep and is located in the north of the Aragón region at 680 m above sea level, with an annual average rainfall of 670 mm per year. Its reproductive management consists of four 45-day-long mating periods per year. As the two farms had very similar characteristics, both in management and type of production, and the lambs were slaughtered at the same weight and age, and at the same time of year, the data obtained from the studied lambs in both farms were pooled and analysed together.

### 2.2. Microbiological Analysis

Microbiological analysis was performed on ten samples of intestinal content. The collected samples were refrigerated and brought to the Exopol laboratory within 24 h. The microbiological samples were surface plated onto blood agar (tryptic soy agar containing 5% sheep red blood cells) (BA) plates (Oxoid PB 5039A) and incubated in aerobic and microaerobic conditions (5–12% CO_2_) for 48 h at 37 °C. Identification of isolates was carried out by matrix-assisted laser desorption/ionisation time of flight (MALDI-TOF, Bruker Daltonics, Bremen, Germany).

### 2.3. Biomolecular Analysis

The ten samples of intestinal content were also subjected to a molecular study for detection of *Clostridium perfringens* by real-time PCR (qPCR). Further, 43 whole blood samples were submitted to the laboratory for the detection of hemoparasites by qPCR. Twenty-one blood samples were from lambs with jaundiced carcasses and 22 from unaffected lambs. Blood samples were primarily analysed for piroplasmas (*Babesia* sp. and *Theileria* sp.), *Mycoplasma ovis* and *Anaplasma* sp. Once a positive result for *Anaplasma* sp. was obtained, a specific *A. ovis* qPCR was performed on these samples. The specific detection of *Anaplasma ovis* was carried out by using the commercial kit EXOone *Anaplasma ovis* (EXOPOL S.L.) and followed the manufacturer’s instructions. This qPCR assay has an analytical sensitivity of 50 copies of genomic equivalent/reaction and includes a quantified synthetic positive control. The assay targets the single copy MSP4 gene that is reported to allow a specific differentiation of *Anaplasma ovis* from the closely related *Anaplasma marginale* [13]. Additionally, an endogenous control was also included in all of these assays in order to avoid false-negative results. The bacterial load was expressed using the quantification cycle (Cq), which is the cycle number where the PCR amplification curve intersects the threshold line [14]. The Cq value can be used to quantify or to determine the presence/absence of the target sequence.

The commercial kit, MagMAX™ Pathogen RNA/DNA (Thermo Fisher Scientific, Austin, TX, USA) with an automated magnetic particle processor (KingFisher Flex System, Thermo Fisher Scientific, Vantaa, Finland) was used for nucleic acids extraction according to the manufacturer’s instructions. Amplification was carried out in a 7500 fast Real-Time PCR machine (Applied Biosystems, Marsiling, Singapore) and results analysed with the respective software (7500 software v2.3, Foster, CA, USA).

### 2.4. Clinical Examination

Clinical examination was performed in 492 lambs one or two days before slaughtering within the five weeks that the study lasted. During the examination, several clinical parameters were recorded: body condition, rectal temperature, the colour of mucous membranes and respiratory, digestive or locomotor clinical signs. Pictures of the mucous membranes and ocular sclera were taken from all studied animals.

### 2.5. Haematological and Biochemical Analysis

Samples of blood with anticoagulant (EDTA) and without anticoagulant were collected from the jugular vein through a vacutainer system from seventy-three of the studied animals (53 from Farm A and 20 from Farm B). These lambs were selected for presenting symptoms such as hyperthermia or pale mucous membranes. Haematology was performed using an automatic haematological counter IDEXX Procyte Dx (IDEXX laboratories, Westbrook, ME, USA). Measured parameters were leucocytes (K/µL), erythrocytes (M/µL), haemoglobin (g/dL), haematocrit (%), platelets (K/µL), MCV (Mean Corpuscular Volume; fL), MCH (mean corpuscular haemoglobin; pg), MCHC (mean corpuscular haemoglobin concentration; g/dL) and reticulocytes (K/µL). Moreover, neutrophils (K/µL), lymphocytes (K/µL), monocytes (K/µL), basophils (K/µL) and eosinophils (K/µL) were analysed in the white blood cells. Biochemical analysis was also performed with sera from five affected animals using an IDEXX Catalysis One device (IDEXX laboratories, Westbrook, ME, USA). Parameters studied were: glucose (mg/dL), creatinine (mg/dL), urea (mg/dL), phosphorus (mg/dL), calcium (mg/dL), total protein (g/dL), albumin (g/dL), globulin (g/dL), alanine aminotransferase enzyme (U/L), alkaline phosphatase enzyme (U/L), gamma-glutamyl transferase (U/L), total bilirubin (mg/dL) and cholesterol (mg/dL).

### 2.6. Pathological Study

Four hundred and sixty-two out of 492 studied animals were followed and observed after slaughtering at the abattoir. There, data on the colour and aspect of the carcass (*n* = 462) and the size of the spleen (*n* = 280) were recorded. In addition, spleen and gastrointestinal tract from 22 of those lambs affected with splenomegaly and showing an intense yellow colour of the intestinal content were taken to the Pathological Service of the Veterinary Faculty of Zaragoza for a more in-depth analysis. For histopathology, tissue samples were fixed in 10% buffered formalin and routinely embedded in paraffin inclusion. Four-µm sections were stained with haematoxylin-eosin.

### 2.7. Statistical Analysis

Descriptive statistics based on counts and proportions were used to define qualitative variables (carcass jaundice and *A. ovis* qPCR results). Analysis of the haematological parameters was performed by non-parametric tests except for monocytes and platelet recounts, which were analysed by Student’s T-test, and the results were shown as mean ± standard error. Carcass jaundice and *A. ovis* qPCR results were used as grouping variables for haematological parameters. In addition, the differences between groups were analysed according to the Mann-Whitney U test in the variables that did not meet the normality criteria. These results were shown as median values. For all cases, *p* < 0.05 was required to consider statistically significant differences. The study was developed with IBM SPSS Statistics V.24 software (IBM, Armonk, NY, USA).

## 3. Results and Discussion

During the lambing period, the studied lambs showed no sign of disease and displayed normal growth for this breed. Mortality during the rearing period was in stock values, not exceeding 5%. They were apparently healthy lambs that after slaughter showed jaundiced carcasses that led to their condemnation. In a first step, the possibility that carotenoid pigments were staining the carcass was discarded. Subsequently, microbiological, molecular and serological analyses were performed in order to diagnose the disorder among the diseases causing jaundice in sheep such as yellow lamb disease caused by *Clostridium perfringens* Type A [15], piroplasmosis by *Babesia* sp. and *Theileria* sp. [16], mycoplasmosis by *Mycoplasma ovis* [17] and anaplasmosis caused by *Anaplasma ovis* [1]. Leptospirosis by *Leptospira* sp. [18] and chronic copper poisoning was dismissed owing to the age of the lambs, the low severity of the process and the anamnesis (the lambs came from different origins). In the study of the intestinal content using PCR the presence of *Clostridium perfringens* was found in a very low quantity (Cq: 32.2 to 36; above 38 is classified as negative), which did not justify the pathological process. Further, detection of *Clostridium perfringens* toxins was negative for alpha, beta and epsilon toxins. Finally, all the affected animals tested negative to *Babesia* sp., *Theileria* sp. and *Mycoplasma ovis*, and positive to *Anaplasma* sp. and *Anaplasma ovis* by qPCR.

### 3.1. Anaplasma ovis Detection by qPCR

Thirty-seven of the 43 analysed lambs were positive to *A. ovis* PCR (86.04%). All tested lambs with icteric carcasses showed positive PCR (21/21), although 72.72% (16/22) of the unaffected lambs also tested positive. However, the bacterial load, expressed in quantification cycles (Cq), was significantly higher in the animals that showed jaundiced carcasses (25.00 vs. 26.16; *p* = 0.004). It has been reported that the mere presence of the bacteria in the blood is not indicative of disease since once the immune system has defeated the illness, the animals remain carriers for life [19]. However, an increase in the bacterial load in blood is observed when the clinical signs appear [6].

Moreover, all the tested lambs that showed severe anaemia were PCR positive (20/20) and none negative lambs had anaemia (6/6). In addition, 17 PCR positive lambs also showed normal blood values. The total erythrocyte count (13.12 vs. 8.63; *p* = 0.002), haematocrit (36.50 vs. 30.53; *p* = 0.031) and haemoglobin (11.80 vs. 9.90; *p* = 0.002) were significantly higher in negative lambs compared to *A. ovis* PCR positive animals. While the MCV (27.70 vs. 35.10; *p* = 0.15) and the reticulocytes (4.45 vs. 9.90; *p* = 0.083) were lower, significant differences were not found (Table 1). Jiménez et al. [6] reported, in an experimental infection carried out with *A. ovis*, that the bacteraemia peak coincided with the lowest erythrocyte count. Subsequently, the bacteraemia decreased and maintained a residual and constant bacterial load in the blood until the end of the study [6].

### 3.2. Clinical Examination

Clinical examination performed before slaughtering showed slight hyperthermia (40.51–41.10 °C) and paleness of mucous membranes in some lambs. No other clinical signs were observed. Fever spikes is one of the symptoms associated with anaplasmosis in sheep [1] and cattle [3].

### 3.3. Haematological Analysis

Lambs that presented icteric carcasses showed severe anaemia with significantly lower erythrocyte count (7.18 vs. 11.97; *p* < 0.001), haematocrit (26.89 vs. 34.82; *p* < 0.001) and haemoglobin (8.50 vs. 11.10; *p* < 0.001) than unaffected lambs (Table 2). Anaemia was regenerative, thus reticulocyte count (18.80 vs. 5.65; *p* = 0.001) and MCV (29.45 vs. 34.50; *p* = 0.001) were also significantly increased in affected animals, although MCV was within the normal range in all lambs (Table 2). Platelet count did not show any difference between groups (442.7 vs. 467.5; *p* = 0.475), although five affected animals showed thrombocytopenia (13.51%). In ovine anaplasmosis, at the beginning, anaemia is normocytic and normochromic, and as the disease evolves, the reticulocyte count increases and anaemia becomes macrocytic and normochromic [6].

*Anaplasma* infects new erythrocytes by a non-lytic mechanism [10]. Thus, anaemia that follows infection is a consequence of the immune response triggered by anaplasma bacteria. Activated macrophages are responsible for the destruction of infected erythrocytes due to opsonisation and nitric oxide production, respectively [12]. In the present study, although monocytosis was observed in almost all affected lambs (35/37, 94.59%; with an average value of 2.10 ± 0.146), it was also detected, although to a lesser extent, in unaffected animals (29/34, 85.29%; with an average value of 1.71 ± 0.149). However, no significant differences were found between them. Lastly, the other white blood cells were within normal ranges.

Biochemical analysis indicated that the analysed animals had increases in alkaline phosphatase enzyme (ALP; 4/5), gamma-glutamyl transferase (GGT; 5/5) and total bilirubin (4/5). Anaemia can be the cause of liver failure as a consequence of hepatocellular hypoxia, which would explain the elevated serum levels of the enzymes ALP and GGT. However, it should be noted that an increase in ALP levels in young animals during their growth periods has been described [20].

### 3.4. Pathological Findings

**Figure 1:**
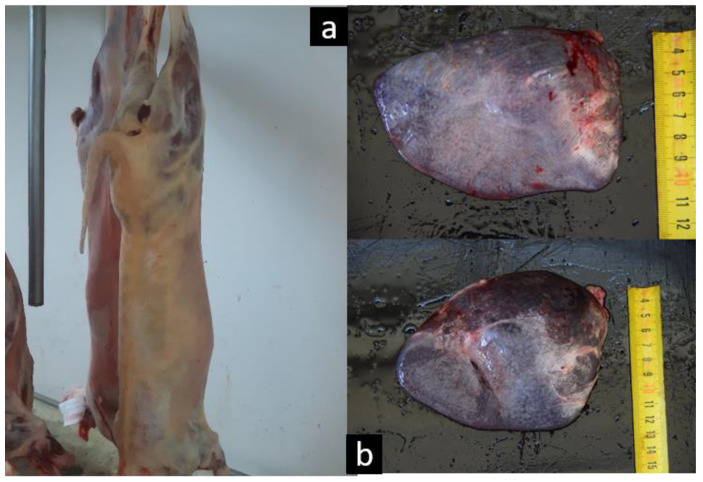
Jaundiced carcass detected at the abattoir (**a**) and spleens from two of the affected animals showing an evident splenomegaly (**b**).

After slaughter, 161 of 462 studied lambs (34.84%) showed jaundiced carcasses (Figure 1a), and 79.64% (223/280) presented splenomegaly at the abattoir (Figure 1b); 92 had large or very large spleens (32.85%), and 131 (46.78%) had a slight increase in size. Although jaundice is a clinical sign associated with severe episodes of haemolysis, ovine anaplasmosis—in contrast to other tick-borne diseases—has generally been described as a mild and chronic process in which haemolysis fails to produce clinical signs such as jaundice or haemoglobinuria, with only anaemia and weaknesses being observed [3,5,21].

Grossly, spleens of the 22 lambs that underwent a more in-depth pathological study were markedly enlarged (average weight 223.43 g vs. 60.00 g in healthy). Additionally, trichobezoars were found in the abomasum, and a variable degree of jaundice in the mesenteric fat was observed, and intestinal content constantly showed an orange discolouration at the ceca. Microscopically, the splenic red pulp showed a diffuse increased cellularity, mainly lymphocytes and macrophages admixed with variable quantities of neutrophils and plasma cells. There was moderate to severe hyperplasia of splenic white pulp with activation of germinal centres of lymphoid follicles and increased number of antigen-presenting cells. Hyperplasia of macrophages in the red pulp has been described in chronic haemolytic diseases [22] and may result in the splenomegaly observed in the studied lambs. This splenic change is caused by the production of macrophages to destroy the infected erythrocytes [11,22].

## 4. Conclusions

There are very few studies carried out on clinical anaplasmosis in sheep in the world, and infection with *Anaplasma ovis* has often been neglected, as it has been considered to induce only mild clinical symptoms and thus to be of minor economic importance [5]. In recent years, due to the increase in temperature that accompanies climate change, vector-borne diseases are emerging strongly. In 2014, clinical anaplasmosis in sheep was reported for the first time in Spain causing a severe outbreak [6], and since then the number of cases diagnosed year after year has been increasing. This article describes a new disorder caused by *Anaplasma ovis* that is producing significant economic losses associated with the carcass condemnation of apparently healthy lamb. The authors would like to draw attention to this emerging disease, which is beginning to cause significant economic losses in Spanish sheep farming.

## Figures and Tables

**Table 1:** Haematological parameters (median value and 25th and 75th percentile values) of *A. ovis* PCR negative and positive lambs (threshold value < 38).

Haematological Parameters	PCR Negative (*n* = 6)	PCR Positive (<38) (*n* = 37)	Haematological Threshold Values
Erythrocytes (M/µL)	13.12 ^a^ (12.395–13.440)	8.63 ^b^ (6.800–11.540)	9.49–15.12 M/μL
Haematocrit (%)	36.50 ^a^ (34.75–38.00)	30.53 ^b^ (25.50–36.00)	27.0–42.0%
Haemoglobin (g/dL)	11.80 ^a^ (11.45–12.00)	9.90 ^b^ (7.70–11-10)	10.0–14.9 g/dL
MCV (fL)	27.70 ^a^ (25.30–30.73)	35.10 ^b^ (29.20–39.50)	24.4–32.5 fL
Reticulocytes (K/µL)	4.45 (3.48–11.23)	9.90 (4.85–24.40)	0.0–15.0 K/μL

^a,b^ Different letters within a row indicate significant differences (*p* < 0.05) between PCR positive and negative animals.

**Table 2:** Haematological parameters (median value and 25th and 75th percentile values) of lambs that presented icteric carcasses. Mean ± Standard error of platelets.

Haematological Parameters	Normal Carcass (*n* = 34)	Icteric Carcass (*n* = 37)	Haematological Threshold Values
Erythrocytes (M/µL)	11.97 ^a^ (10.335–13.153)	7.18 ^b^ (6.380–8.515)	9.49–15.12 M/μL
Haematocrit (%)	34.82 ^a^ (31.00–37.00)	26.89 ^b^ (22.00–31.00)	27.0–42.0%
Haemoglobin (g/dL)	11.10 ^a^ (10.30–11.80)	8.50 ^b^ (7.20–9.45)	10.0–14.9 g/dL
MCV (fL)	29.45 ^a^ (25.88–34.73)	34.50 ^b^ (31.20–38.65)	24.4–32.5 fL
Reticulocytes (K/µL)	5.65 ^a^ (4.150–9.525)	18.80 ^b^ (5.400–40.000)	0.0–15.0 K/μL
Platelets (K/µL)	467.5 ± 24.68	442.7 ± 24.28	301–922 K/μL

^a,b^ Different letters within a row indicate significant differences (*p* < 0.05) between those animals with icteric and normal carcasses.
